# Hydroalcoholic extract of *Achillea millefolium* improved blood glucose, liver enzymes and lipid profile compared to metformin in streptozotocin-induced diabetic rats

**DOI:** 10.1186/s12944-020-01228-4

**Published:** 2020-04-27

**Authors:** Shahla Rezaei, Fatemeh Ashkar, Farhad Koohpeyma, Marzieh Mahmoodi, Maryam Gholamalizadeh, Zohreh Mazloom, Saeid Doaei

**Affiliations:** 1 Student Research Committee, Department of Clinical Nutrition, School of Nutrition and Food Sciences, Shiraz University of Medical Sciences, Shiraz, Iran; 2 Shiraz Endocrinology and Metabolism Research Center, Shiraz University of Medical Sciences, Shiraz, Iran; 3 Students Research Committee, Cancer Research Center, Shahid Beheshti University of Medical Sciences, Tehran, Iran; 4 Department of Nutrition, School of Health and Nutrition, Shiraz University of Medical Sciences, Shiraz, Iran; 5 Research Center of Health and Environment, Guilan University of Medical Sciences, Rasht, Iran

**Keywords:** *Achillea millefolium*, Blood glucose, Lipid profile, Liver enzymes, Diabetes

## Abstract

**Background:**

Recent studies have reported that herbal extracts may have some protective effect against the complications of diabetes mellitus. This study aimed to investigate the effects of *Achillea millefolium hydroalcoholic* extract in comparison to metformin on liver damage, lipid abnormality, and glycemic control in diabetic rats.

**Methods:**

Rats were randomly assigned to 7 groups of 10 animals. Diabetes was induced by injection of streptozotocin (STZ) to 4 groups of rats. Three groups of diabetic rats were given 250 mg/kg/day metformin, 25 mg/kg/day *Achillea millefolium* hydroalcoholic extract, or 100 mg/kg/day of this extract. Two non-diabetic groups were also given either 25 mg/kg/day or 100 mg/kg/day *Achillea millefolium* extract. Normal control and diabetic control rats received 1 mL/day of normal saline. Treatments were administered through oral gavage for 28 days. At the end, rats were anesthetized with ether and their serum samples were separated in order to measure blood glucose, serum total protein, lipids, and liver enzymes.

**Results:**

There was a significant reduction in blood glucose, serum liver enzymes, triglycerides, and total- and LDL-cholesterol levels of the *Achillea millefolium* extract-treated groups compared to the other groups. In addition, there was a significant increment in body weight and HDL-cholesterol serum level in the *Achillea millefolium-*treated groups.

**Conclusion:**

*Achillea millefolium* extract compared to metformin reduces lipid abnormality, blood glucose and liver enzymes in STZ-induced diabetic rats. Future clinical studies are warranted to confirm our experimental findings in humans.

## Background

Diabetes mellitus (DM) is one of the most common chronic metabolic abnormalities worldwide caused thousands of deaths yearly, and becoming a growing concern for both western and developing nations [1]. There was a global prevalence of 425 million people with diabetes in 2017, which is expected to rise to 629 million by 2045 [2]. Various forms of DM have widely been identified whereas type 2 DM is the most common and possessed more than 85% of all diabetic cases. Herbal drugs are used as alternatives for routine drugs for diabetic patients [3]. One of these herbal drugs is derived from *Achillea millefolium* belonging to the Asteraceae family and possessed a variety of pharmaceutical benefits, traditionally used in the amelioration of diabetes, high blood pressure, renal stones, muscle pain, acne, and bleeding [3, 4]. These effects are attributed to essential compounds in Achillea species such as tanene, terepen, acetylen, lacton and razin. Additionally, it is reported that the plant Achillea may counteract the side effects of drugs and improve the potency of therapeutic procedures [5].

*Achillea millefolium* attenuates inflammation and related signaling pathways [6]. A recent study reported that *Achillea millefolium* extract improves inflammation by reducing inflammatory cytokines such as IL-1B [7]. Furthermore, it has been reported that Achillea extract decreased lipid peroxidation and improved antioxidant enzyme levels such as glutathione level due to owing to its considerable antioxidant capacity. Hence, this extract can have a protective effect against oxidative stress development and organ damages [8].

In addition, it has antibacterial, antimicrobial, immunological, anti-proliferative, and antiplatelet activity [9]. To a better understanding of molecular mechanisms of Achillea extract against metabolic abnormalities, this study aimed to investigate the effects of hydro-alcoholic extract of *Achillea millefolium* on lipid profile, blood glucose, body weight, and serum liver enzymes in streptozotocin (STZ)-induced diabetic rats.

## Materials and methods

### Experimental animals

For this study 70 male Sprague-Dawley rats (weighing 200–300 g) were obtained from the Laboratory Animals Research Center (Shiraz University of Medical Sciences, Iran). The animals were adapted in animal laboratory for 2 weeks prior to the experiments, and were fed a rat chow diet (Pars Dam Co., Tehran, Iran). Food and drinking water were available ad libitum during the study. Rats were kept in stainless steel cages in a temperature-controlled (22–25 °C) environment. Lighting (12 h light/dark cycles) and humidity (55%) conditions were also controlled. The protocol was approved by the Ethics Committee of Shiraz University of Medical Sciences (Code: 92–01-01-6869).

### Extract preparation

*Achillea millefolium* plant was rinsed, and softly dried at room temperature. Plant materials (300 g) were crashed and then the extract was taken up using percolation approach in 1000 ml of 70% ethanol at room temperature for 72 h. After filtration, ethanol was removed at 40 °C in a rotary and the prepared extract was kept at − 20 °C. Finally, solvent evaporation was performed by vacuum desiccator for 24 h [10].

### Induction of diabetes

In the present study, diabetes was induced intra-peritoneally in overnight-fasted male Sprague-Dawley rats through injection of 60 mg/kg body weight freshly prepared STZ (Sigma, USA) dissolved in a 0.1 mol/L citrate buffer (pH 4.5) [11]. A glucometer (Accu-Chek Active, Roche, Germany) was used to estimate blood glucose levels. The stable blood glucose concentrations were measured 7 days after STZ injection. Blood glucose levels above 300 mg/dl were considered as criteria for diagnosis of diabetes.

### Experimental design

In this experimental study, rats were randomly divided into 7 groups of 10 rats each. One group were control rats that received 1 mL/day normal saline (normal control). Two groups were non-diabetic rats treated with either 25 mg/kg/day or 100 mg/kg/day *Achillea millefolium* hydroalcoholic extract. The other 4 groups were induced by STZ and then received 1 mL/day normal saline (diabetic control), 250 mg/kg/day metformin, 25 mg/kg/day, or 100 mg/kg/day *Achillea millefolium* hydroalcoholic extract. Normal saline or *Achillea millefolium* extract was administered through oral gavage. The treatment period was 28 days.

### Biochemical parameters

Rats were monitored weekly for body weight and blood glucose. On day 28 of the intervention, the rats were anesthetized after 12 h fasting. Blood samples were collected by cardiac puncture, and serum was separated by centrifugation at 3500 rpm for 15 min. Serum samples were stored at − 80 °C until analysis. Lipid profile (triglycerides and total-, LDL, and HDL-cholesterol) were evaluated using commercially available kits (Pars Azmoon Co., Iran).

### Statistical analysis

Statistical analysis was performed using SPSS software version 22.0. The data are presented as means ± standard deviation (SD). Paired samples T-Test were used to compare the means of blood glucose and body weight changes. One-way ANOVA was used for other parameters with LSD as the post-hoc test. The results were considered to be significant when the *P*-values were < 0.05.

## Results

### Effect of *Achillea millefolium* on body weight

**Fig. 1:**
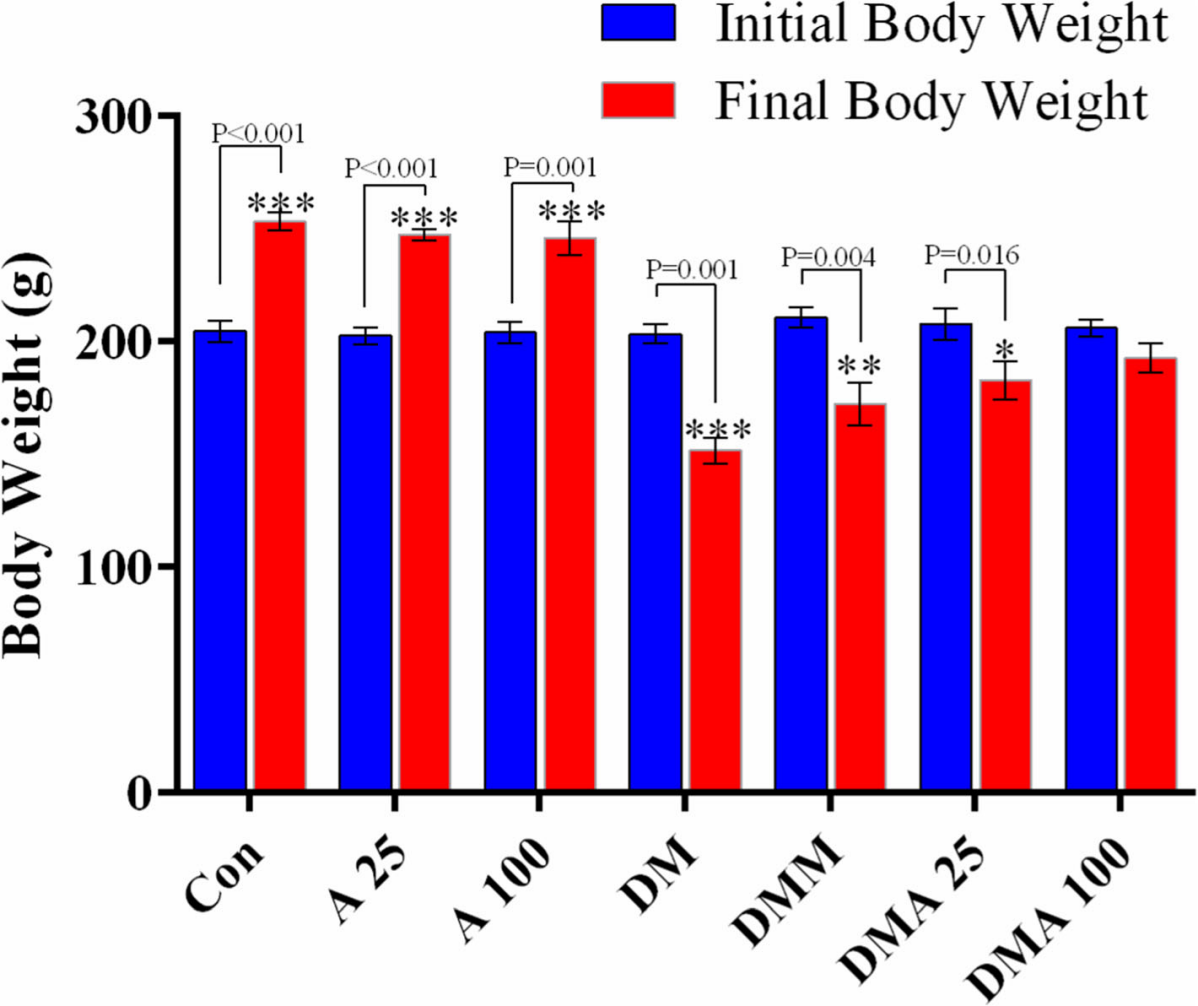
Evaluation of body weight in experimental groups. Con: healthy control group; A25: healthy *Achillea millefolium* extract 25 mg/kg group; A100: healthy *Achillea millefolium* extract 100 mg/kg group; DM: Diabetes mellitus group; DMM: Diabetes mellitus+metformin 250 mg/kg group, DMA25: Diabetes mellitus + *Achillea millefolium* extract 25 mg/kg group, DMA100: Diabetes mellitus + *Achillea millefolium* extract 100 mg/kg group. *p < 0.05, **p < 0.01, ***p < 0.001

There was a significant increase in body weight in healthy groups receiving *Achillea millefolium*. Mean body weight was significantly reduced in the diabetic group compared to the baseline (Fig. 1). There was a significant decrease in body weight in diabetic groups receiving *Achillea millefolium* and metformin compared to the baseline. However, high dose of *Achillea millefolium* (100 mg/kg) increased body weight compared to the baseline (*P* < 0.05).

### Effect of *Achillea millefolium* on blood glucose

**Fig. 2:**
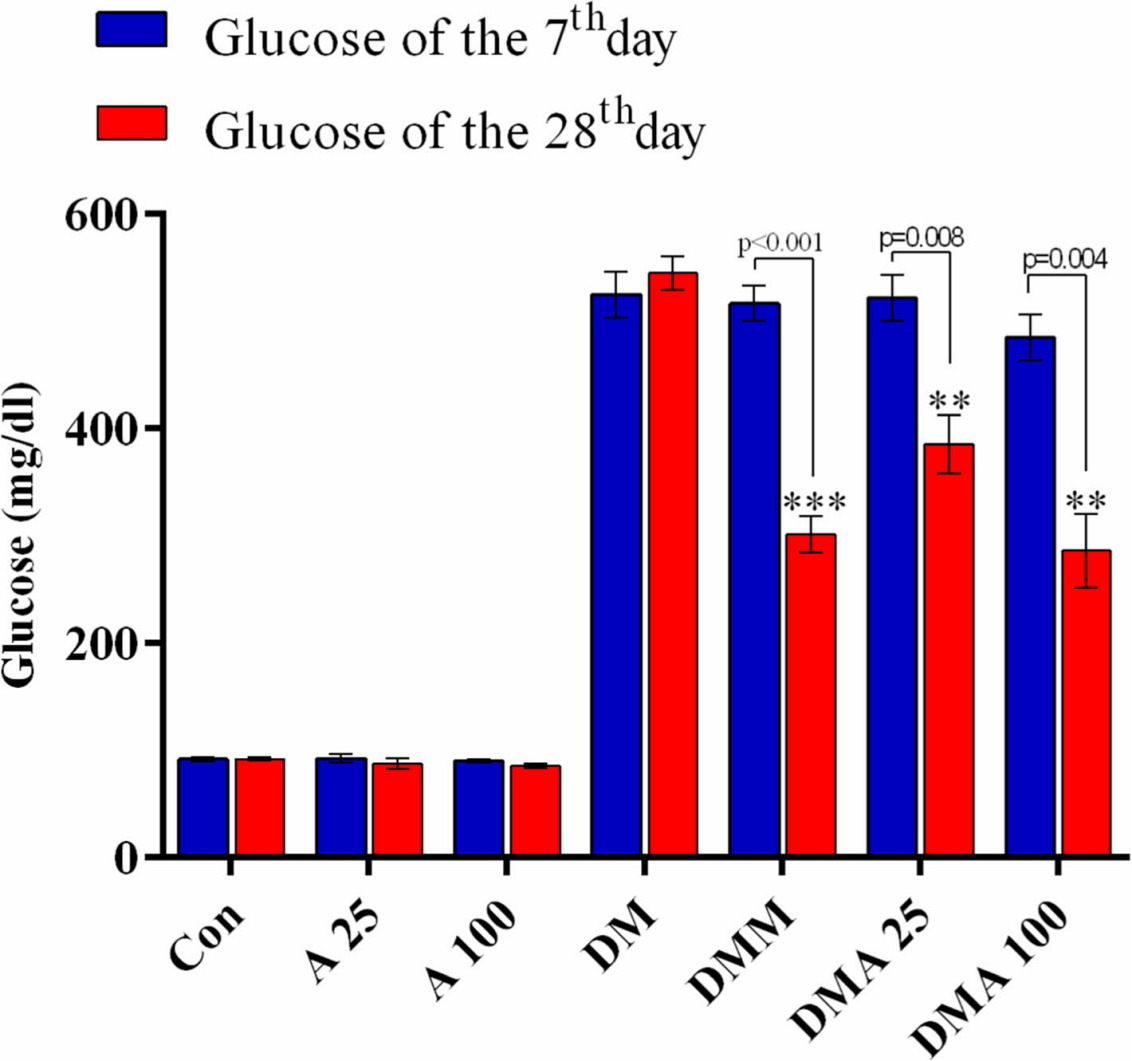
Evaluation of fasting blood glucose in experimental groups. Con: healthy control group; A25: healthy *Achillea millefolium* extract 25 mg/kg group; A100: healthy *Achillea millefolium* extract 100 mg/kg group; DM: Diabetes mellitus group; DMM: Diabetes mellitus + metformin 250 mg/kg group, DMA25: Diabetes mellitus + *Achillea millefolium* extract 25 mg/kg group, DMA100: Diabetes mellitus + *Achillea millefolium* extract 100 mg/kg group. **p < 0.01, ***p < 0.001

STZ significantly increased blood glucose compared to the non-diabetic groups (Fig. 2). There were significant reductions in blood glucose of groups treated with either metformin or *Achillea millefolium* extract compared to the diabetic control. The decrease was in the same extent in the metformin- and 100 mg/kg *Achillea millefolium*-treated groups.

### Effect of *Achillea millefolium* on serum liver enzymes

**Fig. 3:**
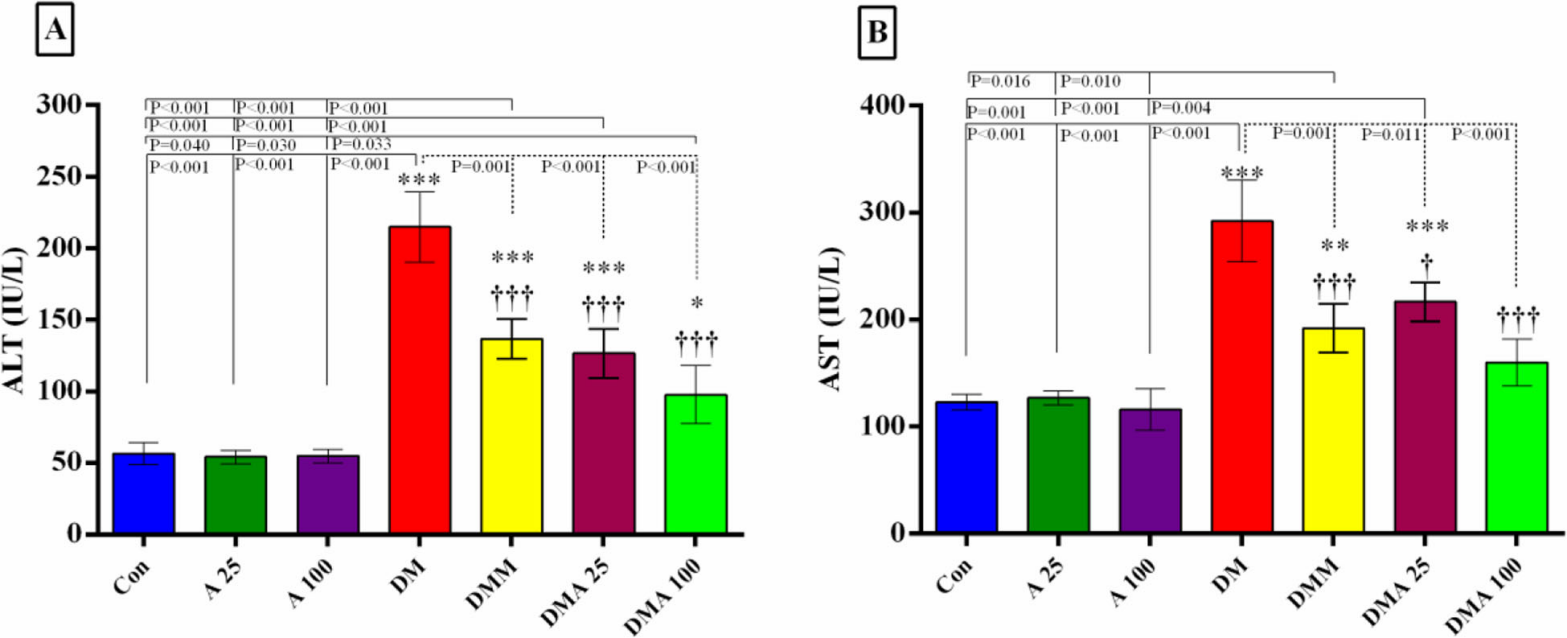
Evaluation of liver enzymes in experimental groups. Graph **a**, **b**; Con: healthy control group; A25: healthy *Achillea millefolium* extract 25 mg/kg group; A100: healthy *Achillea millefolium* extract 100 mg/kg group; DM: Diabetes mellitus group; DMM: Diabetes mellitus + metformin 250 mg/kg group, DMA25: Diabetes mellitus + *Achillea millefolium* extract 25 mg/kg group, DMA100: Diabetes mellitus + *Achillea millefolium* extract 100 mg/kg group. *p < 0.05, Con vs.DM, DMM, DMA25, and DMA100 groups. †*p* < 0.05, DM vs. DMM, DMA25, and DMA100 groups. $ *p* < 0.05 DMA25 vs DMM and DMA100 groups. *p < 0.05, †*p* < 0.05, **p < 0.01, ***p < 0.001, †††*p* < 0.001

STZ significantly increased serum concentrations of liver enzymes, ALT and AST, compared to the non-diabetic groups (Fig. 3a and b). There were significant reductions in ALT and AST concentrations in the metformin- and *Achillea millefolium*-treated groups compared to the diabetic group.

### Effect of *Achillea millefolium* on serum lipid profile

**Fig. 4:**
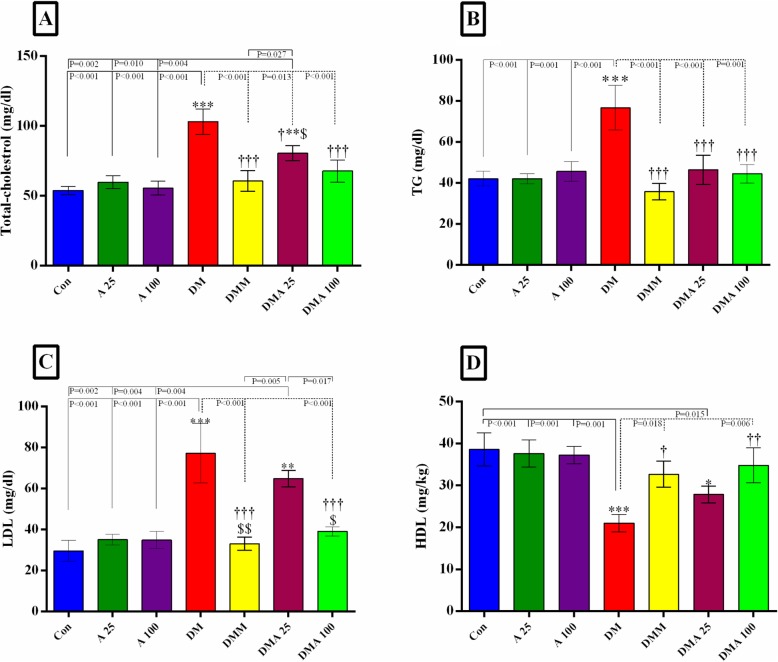
Evaluation of lipid profile in experimental groups. Graph **a**-**d**: Con: healthy control group; A25: healthy *Achillea millefolium* extract 25 mg/kggroup; A100: healthy *Achillea millefolium* extract 100 mg/kg group; DM: Diabetes mellitus group; DMM: Diabetes mellitus + metformin 250 mg/kg group, DMA25: Diabetes mellitus + *Achillea millefolium* extract 25 mg/kg group, DMA100: Diabetes mellitus + *Achillea millefolium* extract 100 mg/kg group. *p < 0.05, Con vs.DM, DMM, DMA25, and DMA100 groups. †*p* < 0.05, DM vs. DMM, DMA25, and DMA100 groups. $ *p* < 0.05 DMA25 vs DMM and DMA100 groups. *p < 0.05, †*p* < 0.05, $ *p* < 0.05. **p < 0.01, ††*p* < 0.01, $$ *p* < 0.01. ***p < 0.001, †††*p* < 0.001

STZ significantly increased serum total- and LDL-cholesterol and TG compared to the non-diabetic groups (Fig. 4a to d). Metformin and *Achillea millefolium* significantly decreased total cholesterol and TG but only metformin and the dose of 100 mg/kg *Achillea millefolium* were effective for LDL- and HDL-cholesterol while 25 mg/kg *Achillea millefolium* did not significantly improve LDL- and HDL-cholesterol.

## Discussion

In this study, we assessed the effect of 25 mg/kg/day and 100 mg/kg/day *Achillea millefolium* hydroalcoholic extract in STZ-induced diabetic rats. The results identified that this extract had a beneficial effect on serum glucose, lipids, and liver enzymes compared to metformin treated groups and the controls. These effects were also more pronounced in 100 mg/kg/day dose compared to 25 mg/kg/day.

Sadeghi et al. reported similar results from *Achillea wilhelmsii* on blood glucose in male rats [3]. In another study, a similar effect for *Achillea millefolium* extract was also obtained [12]. It is well known that hyperglycemia in diabetes mellitus exacerbates oxidative stress [13]. Oxidative stress generates a depletion in antioxidant capacity, glutathione level and NADPH level. Also, protein oxidation and glycated proteins produced in this condition exacerbate damage in various organs and it has been reported that antioxidants can ameliorate these changes [14]. According to several studies, this plant reported antioxidant properties [15–18] and can thus be used for amelioration of complications of oxidative stress conditions like diabetes mellitus [19]. This extract might act as a hypoglycemic factor and diminish intestinal glucose absorption due to its antioxidant properties. Further studies should evaluate oxidative stress, e.g., by measuring activities of antioxidant enzymes including SOD and GSH-P, and levels of MDA - an indicator of lipids peroxidation.

Our results found that STZ led to a significant increase in serum levels of total cholesterol, TG and LDL-cholesterol and decrease in HDL-cholesterol. In the present study, a considerable reduction in serum total cholesterol, TG and LDL-cholesterol and a significant increase in HDL-cholesterol in the treated groups was demonstrated, and the dose of 25 mg/kg *Achillea millefolium* appeared to be less beneficial. It has been found that insulin resistance led to increase apo-B secretion. Hence, these changes result in abnormal lipid profile and increase in apo-B lipoprotein plasma level such as LDL, very low density lipoprotein (VLDL) and chylomicron [19, 20]. In addition, higher level of reactive oxygen radicals in diabetes mellitus damage lipid memebrane particularly poly unsaturated fatty acids construction and caused an abnormal lipid metabolism and lipid peroxidation [14, 20]. Abnormal elevated lipid level ameliorates antioxidant capacity and increased oxidative stress level that caused various organ damage [21].

Asgary et al., reported that Achillea *wilhelmsii* extract preserved SH groups of products in antioxidant enzymes and membrane. Thus, this extract prevented from oxidative stress and membrane damage through lipid peroxidation. Because of these properties, this extract had antioxidant and anti-inflammatory effects [19, 18]. Similar results reported in one study by Asghary et al. about protective effect of Achillea on abnormal lipid profile [22].

STZ caused a considerable increase in serum levels of liver enzymes while treatment with metformin or *Achillea millefolium* extract significantly mitigated these elevations. Augmentation of hepatic enzymes by STZ is likely due to liver damage caused by oxidative stress resulting from hyperglycemia [23]. However, we could not measure the antioxidant potential of *Achillea millefolium* and *Achillea santolina* extracts in protect liver from STZ-induced oxidative stress, but it is evidenced by decreased malondialdehyde and protein carbonyls and improved levels of antioxidant enzymes superoxide dismutase and catalase [23].

In contrast to the observed hepatoprotective effect of *Achillea millefolium* and *Achillea santolina*, Hosbas et al. reported that doses of 250–750 mg/kg/day *Achillea biebersteinii* ethanol extract failed to reduce ALT and AST plasma levels in carbon tetrachloride-induced liver damage in rats [24]. The higher doses of the extract (750 mg/kg/day) used in their study might have left toxic effects on liver, leading to increased liver enzymes.

Our results identified that there was a significant increase in body weight in the treatment groups compared to the diabetic group. Nematy et al. reported that *Achillea millefolium* had positive dose-related effects on appetite in rats [25]. Previous studies found that Flavonoid-rich foods such as some types of honey have beneficial effects on lipid profile of diabetic rats [26]. Flavonoids which are found in *Achillea millefolium* [18] can block serotonin receptors and increase plasma ghrelin concentration [25, 27], and appetite [25, 28]. Because the quality of chemical materials and utilized tools in each experimental assay may differ from another one, therefore further recommendation of this plant extract for clinical applications is needed to be investigated under large-scale in vivo experiments. More importantly, accentuating on this point that plants and their respected metabolites are supplementary agents to management of human diseases should be taken into consideration for clinical studies to get the high-quality results.

## Conclusion

The *A. millefolium* extract in comparison to metformin reduces lipid abnormality, hyperglycemia, and hepatic enzymes with a dose-dependent effect in diabetic rats. The observed results herein will be required to be investigated under larger in vivo studies. Further in-depth studies are warranted to confirm exact molecular mechanisms of these plant extracts in humans.

## Data Availability

Not applicable.

## Abbreviations

ALT: Alanine Aminotransferase
AST: Aspartate Aminotransferase
HDL: High-density lipoprotein
LDL: Low Density Lipoprotein
STZ: Streptozotocin
T2DM: Type 2 diabetes mellitus
VLDL: Very low density lipoprotein
